# Compressive Strength of Temporary Crowns Made from Default design in Two Types of Software

**DOI:** 10.30476/DENTJODS.2022.89736.1433

**Published:** 2023-03

**Authors:** Tahereh Ghaffari, Amin Nourizadeh, Elnaz Shafiei, Farhang Mahboub, Amir Reza Kalantari

**Affiliations:** 1 Dept. of Prosthodontics, Faculty of Dentistry, Tabriz University of Medical Sciences, Tabriz, Iran; 2 Dept. of Prosthodontics, Faculty of Dentistry, Tabriz Medical Sciences, Islamic Azad University, Tabriz, Iran; 3 Postgraduate Student, Dept. of Prosthodontics, Faculty of Dentistry, Tabriz University of Medical Sciences, Tabriz, Iran

**Keywords:** Fixed prosthesis, Temporary crown, Compressive strength, 3Shape Dental System software, Exocad software

## Abstract

**Statement of the Problem:**

Various default values in each software can eventually lead to different crown thicknesses and affect their compressive strength.

**Purpose:**

This study aimed to compare the compressive strength of temporary crowns made with the milling machine, designed in Exocad and 3Shape Dental System software.

**Materials and Method:**

In this *in vitro* study, 90 temporary crowns were made and evaluated based on each software settings. For this purpose, a sound premolar was first scanned as a pre-operative model by 3Shape laboratory scanner. The standard tooth preparation and scanning were performed, and then the temporary crown files (designed by each software) were transferred to the Imesicore 350i milling machine. A total of 90 temporary crowns (45 based on the file of each software) were made using poly methyl methacrylate (PMMA) vita cad-temp block. The value of compressive force displayed on the monitor was recorded at the first crack and the ultimate failure of the crown.

**Results:**

The first crack and the ultimate strength of crowns designed with the Exocad software was 903.5±96N and 1490±139.3N and for crowns designed with 3Shape Dental System software was 1060.4±160.2N and 1691.1±73.9N, respectively. The amount of compressive strength of temporary crowns made with 3Shape Dental System was significantly higher than those made with Exocad software and this difference was statistically significant(*p*= 0.000).

**Conclusion:**

The compressive strength of temporary dental crowns made by both softwares is in a clinically acceptable range, but considering that the average compressive strength in the 3Shape Dental System group was slightly higher than of the other group, it is preferable to design and fabricate with 3Shape Dental System software to increase the compressive strength of these crowns.

## Introduction

The loss of one or more teeth in patients, leads to functional problems such as poor chewing and speech, as well as inappropriate esthetic, self-image destruction, and loss of emotional balance, which subsequently would affect their quality of life [ 1 ]. Increasing patients' demand for maintaining the beauty and function of their dental system has led to the increasing use of dental prosthesis [ 2 ]. Fixed prosthodontics treatments can range from the restoration of a single tooth to full occlusion reconstruction. Replacing lost teeth with fixed prostheses can increase the comfort and ability to chew, ensure the health of dental arches, and in many cases increase the patient's self-confidence [ 3 ]. In addition, using fixed prostheses and providing an optimal occlusion can improve the orthopedic stability of the temporomandibular joint [ 3 ]. Temporary crowns are used as an essential component in treatments, which are based on fixed prosthodontics and implants. A temporary crown has many advantages such as pulp protection, periodontal health, maintaining the position of the tooth and preventing its displacement or the opposite tooth [ 4 ]. A temporary crown generally protects the prepared tooth until the definitive restoration is delivered [ 4 ].

Various methods for prosthetic reconstruction of edentulous spaces or repair and reinforcement of severely damaged teeth have been introduced [ 1 , 5 ]. In general, these methods can be divided into two categories as traditional and digital [ 5 ]. The traditional method is based on the use of conventional impression materials and laboratory procedures such as casting and wax pattern making and so on [ 6 ], which in addition to having more errors, it has a long process of treatment [ 7 ]. The digital method has several benefits such as increasing the adaptation of the definitive prosthesis and patient comfort in the treatment process, reducing prosthesis preparation time and at the same time the risk of human and laboratory errors. It also reduces the cost of treatment [ 8 ]. Digital dentistry consists of three phases including digitizing the teeth and oral environment by intraoral or laboratory scanners, design of restoration or oral appliance in specialized computerized equipment (CAD), and conversion of digital design into physical model (CAM) [ 9 ]. Digital dentistry, in turn has a variety of methods for restoring lost structures using various softwares. The Exocad and 3Shape Dental System are some of the most widely used software in this field. This software offers different components in the design, which can be by default or be changed in any software environment [ 10 ].

The software defaults for designing crown in Exocad and 3Shape Dental System software can lead to different spaces between the inner surface of the crown and the outer surface of the prepared tooth. This can subsequently lead to different crown thicknesses in a standardized anatomy and affect its compressive strength [ 11 ]. Given the lack of sufficient studies in this regard, we decided to study and compare the compressive strength of temporary crowns made with milling machine resulting from the design in two Exocad and 3Shape Dental System softwares, and present the results in this article. The hypothesis of this study was that temporary crowns made from the default design in Exocad software exhibit similar strength as 3Shape Dental System software.

## Materials and Method

This descriptive-interventional study was performed in 2019-2020 at the Dentistry School of Tabriz University of Medical Sciences to evaluate and compare the compressive strength of temporary crowns made with the milling machine designed in Exocad and 3Shape Dental System softwares. 

The sample size was determined by G*Power v.3.1 software based on the results of the pilot study, and the impact size of the compressive strength of temporary crowns made with the milling machine resulting from the design in each software was considered equal to 0.6. Finally, considering the type I error of α = 0.05 and the test power of 80%, the sample size was calculated equal to 90 items (45 samples for each software). Crowns with cracks, structural defects, and lack of structural integrity were excluded from the study.

First, for the preparation of standardized anatomy on the outer surface of the crown, a sound premolar was scanned as a pre-operative model by E3 3Shape laboratory scanner (3shape, Copenhagen, Denmark) to make exactly the shape of the outer surface of the temporary crown in both softwares. Then, using Speedex condensation silicone (Coltene, Altstätten, Switzerland), putty index was made for tooth preparation. A prosthodontist used this index and prepared the tooth with a deep chamfer finish line for a full coverage crown in compliance with principles of tooth preparation (2 mm occlusal clearance and 1.2 mm reduction of buccal and lingual surface). Then, an impression was taken from the prepared tooth with Speedex condensation silicone (Coltene, Altstätten, Switzerland) and a wax pattern was made and poured by VeraBond nickel-chromium alloy (AalbaDent, USA) to fabricate a metal die for testing the samples in the universal testing machine(Hounsfield H5KS, UK). Next, the prepared tooth was scanned by a scanner (3Shape E3 laboratory scanner) and then the scan files were matched together, so that the designed crown can be the exact size of the unprepared premolar tooth. Then, the prepared scan file was transferred to each of the Exocad (GmbH, Darmstadt, Germany) and 3Shape Dental System (3Shape, Copenhagen, Denmark) software to perform the design steps. Upon completion of the design process, the files were transferred to the imesicore 350i milling machine (CORITEC, Eiterfeld, Germany) and a total of 90 temporary crowns (45 crowns based on each software file) were made using the poly methyl methacrylate (PMMA) VITA CAD-temp block (VITA, Bad Säckingen, Germany). The fabricated crowns were investigated by a magnifying glass and gauge in terms of cracks, structural defects, and lack of structural integrity. The prepared samples were tested to compare the compressive strength in the universal testing machine (Hounsfield H5KS, UK) while specimens were placed on a cast metal die and a force of 1mm/min was applied to the central groove of the temporary crowns by a 3 mm diameter round chisel [ 12 ]. The amount of force displayed on the monitor was recorded at the first crack and the ultimate failure of the crown. Finally, after collecting the compressive strength of the samples in the study checklist, the data were statistically analyzed in SPSS software version 22. First, the normal distribution of data was investigated using the Kolmogorov-Smirnov test and normal quantitative data were reported as mean±deviation. Independent t-test (for normal quantitative variables) was used to compare the studied variable. *p* Value of less than 0.05 was considered statistically significant.

## Results

**Figure 1:**
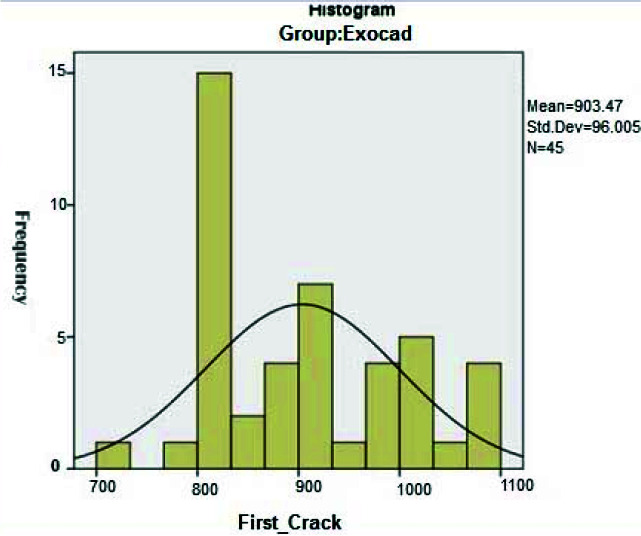
Distribution of required force for the first crack in temporary crowns based on Exocad software

**Figure 2:**
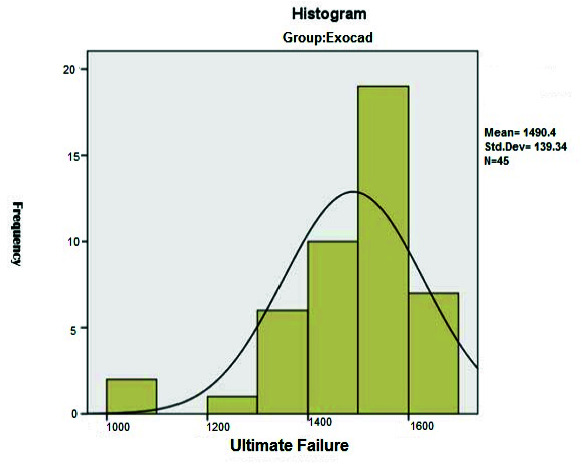
Distribution of required force for the ultimate failure of temporary crowns based on Exocad software

**Figure 3:**
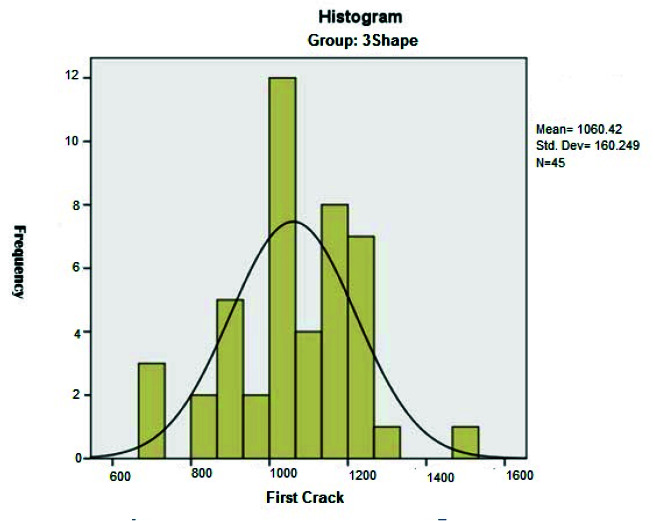
Distribution of the compressive strength for the first crack in temporary crowns based on 3Shape Dental System software

**Figure 4:**
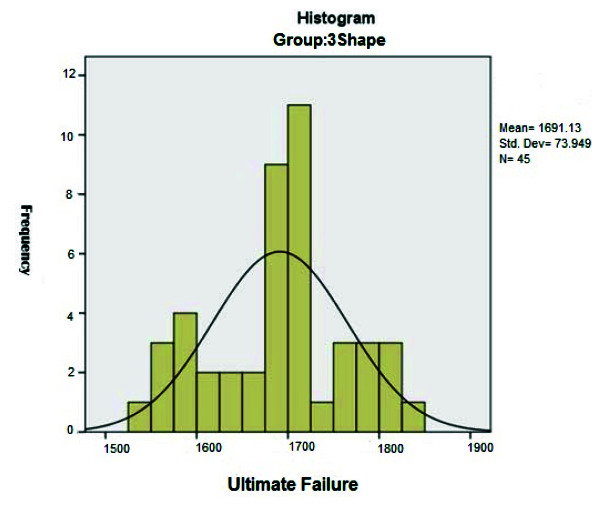
Distribution of the ultimate compressive strength of temporary crowns based on 3Shape Dental System software

Examination of the compressive strength of the studied temporary crowns showed that in the temporary crowns made with the milling machine resulting from design in the Exocad software, the average force required to create the first crack was 903.5±96 N (Figure 1) and for the ultimate failure, it was equal to 1490.4±139.3N (Figure 2). However, for the samples made from design in the 3 Shape Dental System software, the average force required to create the first crack was equal to 1060.4±160.2N (Figure 3) and for the ultimate failure, it was equal to 1691.1±73.9 N (Figure 4).

The independent t-test indicated a significant difference (*p*= 0.000) between the amount of force required to create the first crack and the ultimate failure in temporary crowns made by Exocad and 3Shape Dental System softwares and the latter yielded a significantly higher amount (Table 1).

**Table 1:** Comparison between the mean compressive strength of temporary crowns based on two software systems

Groups	First crack		Ultimate compressive strength	
	Mean	SD	Mean	SD
3Shape Dental	1060.42	160.249	1691.13	73.949
Exocad	903.47	96.005	1490.4	139.34
*p* Value	.000^*^		.000^*^	

## Discussion

The null hypothesis was rejected. It was shown in the present study that the amount of compressive strength of temporary crowns made by 3Shape Dental System software was higher than Exocad software. The 3Shape Dental System is a complete and integrated CAD/CAM for designing prosthetic restorations; this software automatically scans images and completely reconstructs 3D models [ 13 ]. In addition, the 3Shape Dental System has the ability to prepare 3D models based on the type of materials required by technicians, dentists, and patients [ 13 ]. In general, it has been optimized for frameworks, crowns, and bridges fabrication [ 13 ]. The main advantages of this software are doing projects at high speed, the ability to view virtual 3D models without wasting materials and no need for special preparation for the design of crowns [ 13 ].

Each software has its own default values for marginal gap, cement space, and crown thickness and so on. These defaults are stored in the software memory for different employed materials and the type of crown. These assumptions are defined for standard conditions to achieve the best clinical results. It is worth noting that in clinical situations, some of these defaults can be changed and customized depending on the number of abutments, preparation convergence, and possible undercuts [ 10 ].

For example, in 3shape software, a space of a few microns is defined in the one-millimeter strip of the margin area, which causes acceptable compliance of restoration according to the software designers. Moreover, in the same software to avoid interference of the milling machine’s burr, in point angles that the diameter of which is thinner than the milling burr diameter, by default, the drill compensation option is used, which ultimately reduces the wall thickness in that place, to compensate the drill in that small area; and according to the software designer, this extra amount of cutting has a significant effect on matching the inner surface of the crown and teeth in that area [ 15 ]. The cement gap and extra cement gap default standard for manufacturing PMMA provisional crowns in 3shape dental system software is 0.04mm and 0.85mm and in Exocad software is 0.04mm and 0.08mm, respectively [ 15 ]. All temporary crowns that have been tested had the minimum thickness of 0.8mm, which is necessary for provisionals.

The study of Reeponmaha *et al*. [ 14 ], which compared the fracture strength after thermo-mechanical aging between provisional crowns made with CAD/CAM and conventional method showed that provisional crowns fabricated using the CAD/CAM process and the conventionally fabricated bis-acryl resins exhibited significant higher fracture strength compared to conventionally fabricated monomethacrylate resins after the aging regimen. In the study of Al-Hawwaz *et al*. [ 15 ] regarding the internal and marginal adaption of full-contour zirconia crowns made by CAD/CAM using InLab SW, 3Shape Dental System, and Exocad software, it was concluded that the lowest marginal gap was related to InLab SW, Exocad, and 3Shape Dental System software, respectively. In the study of Shembesh *et al*. [ 16 ] on comparing the accuracy of marginal adaption of CAD/CAM restorations by different molding techniques using Exocad software, it was shown that in addition to the software used, the marginal gap also depends on the type of scanning device. The results of Ashtian *et al*. [ 17 ] study, which was about comparing the dimensional accuracy of intracoronal restorations made by traditional and digital methods using 3Shape Dental System software, also stated that in addition to software, the type of manufacturing system is important in the dimensional accuracy of intracoronary restorations.

Although it was shown in the present study that the amount of compressive strength of temporary crowns made by 3Shape Dental System software was higher than Exocad software, but these values are within the clinically acceptable range in both softwares. The maximum biting force (300–600 N) in natural dentition is in the first molar region [ 18 - 20 ]. The values are much higher in patients with bruxism [ 21 ]. Some studies have shown that these values can rise to 720-815 N [ 21 - 22 ].

 Also, considering that changing the default values (decrease or increase) can affect the crown thickness and the degree of adaptation of the prosthesis, these results do not mean that temporary crowns made with 3Shape Dental System software are superior in terms of compressive strength over those made with Exocad software. Finally, previous studies have considered the role of other factors such as the type of scanner and the type of milling system (manufacturing method) to be effective in the quality of fabricated crowns. Given that, to achieve more accurate results on this topic, it is necessary to conduct studies considering factors such as the type of scanner and the milling system. 

It is necessary to denote that the testing in this study was done under static load. Intraorally, teeth are subjected to multiple loading and different thermal environments during mastication and therefore further mouth motion fatigue testing can be done to closely simulate clinical condition. Further studies can be performed with resin cement to evaluate if the cement causes any characteristic change in the mechanical property and the type of fracture of the provisional crowns.

## Conclusion

In the present study, the results showed that the compressive strengths of temporary dental crowns are clinically acceptable by both softwares. However, because the average compressive strength in a standardized anatomy in the 3Shape Dental System group was slightly higher than the other group, it is recommended to design and manufacture temporary dental crowns by using this software. 

## Acknowledgments

This study was conducted with the financial support of Tabriz University of Medical Sciences; this article is based on the approved dissertation No. 64080.

## Conflict of Interest

The authors declare that they have no conflict of interest.
